# SARS-CoV-2 Seroprevalence in Children under 5 Years Old—A Regional Seroepidemiological Study

**DOI:** 10.3390/medicina60030384

**Published:** 2024-02-24

**Authors:** Felicia Trofin, Cătălina Luncă, Diana Păduraru, Dana-Teodora Anton-Păduraru, Elena Roxana Buzilă, Eduard Vasile Nastase, Ancuta Lupu, Vasile Valeriu Lupu, Olivia Simona Dorneanu

**Affiliations:** 1 Department of Preventive Medicine and Interdisciplinarity—Microbiology, “Grigore T. Popa” University of Medicine and Pharmacy, 700115 Iasi, Romania; felicia.trofin@umfiasi.ro (F.T.); elena-roxana.buzila@umfiasi.ro (E.R.B.); olivia.dorneanu@umfiasi.ro (O.S.D.); 2 “Sf. Maria” Children Emergency Hospital, 700309 Iasi, Romania; anca_ign@yahoo.com (A.L.); vasile.lupu@umfiasi.ro (V.V.L.); 3 “Dr. C.I. Parhon” Clinical Hospital, 700503 Iasi, Romania; diana_paduraru@email.umfiasi.ro; 4 Department of Mother and Child Medicine, “Grigore T. Popa” University of Medicine and Pharmacy, 700115 Iasi, Romania; 5 Iasi Regional Center for Public Health, National Institute of Public Health, 700465 Iasi, Romania; 6 Department of Internal Medicine II—Infectious Diseases, “Grigore T. Popa” University of Medicine and Pharmacy, 700115 Iasi, Romania; eduard-vasile.nastase@umfiasi.ro; 7 Clinical Hospital of Infectious Diseases “Sf. Parascheva”, 700116 Iasi, Romania

**Keywords:** SARS-CoV-2, COVID-19, seroprevalence, IgG, children

## Abstract

*Background and Objectives:* The aim of this research was to assess the spread of SARS-CoV-2 infection; the study was motivated by parental hesitancy regarding child vaccination, and the potential passive immunity of infants acquired through breastfeeding from mothers vaccinated against COVID-19 or infected with SARS-CoV-2. *Materials and Methods*: We quantified the anti-SARS-CoV-2 immunoglobulin G (IgG) titer in the serum of 743 children under 5 years old, hospitalized between 1 August 2022, and 15 September 2023. *Results*: Among the participants, 52.76% had an anti-SARS-CoV-2 IgG titer that exceeded the reactivity threshold of the kit used, with an average of 1558.01 U/mL across the entire group. By age-specific categories, SARS-CoV-2 antibody prevalence was 43.04% for 0–12 months, 42.22% for 12–24 months, 61.67% for 24–36 months, 65.17% for 36–48 months, and 68.55% for 48–59 months. Gender analysis revealed 55.32% male participants, with a 52.07% seropositivity rate. Notably, IgG titer correlated positively with the child’s age. Gender, admission diagnosis, and emergency department presentation were not variation factors of the IgG titer. *Conclusions*: The majority of children in the study group demonstrated IgG against SARS-CoV-2, and this rate increased with the child’s age. Also, the IgG titer increased with the child’s age.

## 1. Introduction

As of December 2023, the global incidence of SARS-CoV-2 infection reached a cumulative total of 772,138,818 cases, concurrently accompanied by an anti-COVID-19 vaccination coverage of 66.1% [1].

As of 21 January 2024, data from the National Institute of Public Health in Romania indicated the registration of 3,519,340 cases of COVID-19 within the country. Among these cases, a prevalence rate of 5% has been documented specifically among children aged 0 to 9 years [2]. 

As of early October 2023, data from the European Centre for Disease Prevention and Control (ECDC) indicated that 26.7% of the population below 18 years of age in Europe had received an initial dose of the COVID-19 vaccine, with 24.5% having completed the primary vaccination series, and only 1.3% had received the initial booster vaccination. Moreover, restricting our examination to the demographic cohort of 0–4 years, the ECDC documents a vaccination coverage of 0% on a continental scale, while for the age group spanning 5–9 years, the ECDC reports a vaccination coverage of 14.2% [3].

On 31 December 2023, the National Center for Supervision and Control of Communicable Diseases in Romania documented that 10,089 children had received a singular dose of the Pfizer pediatric anti-COVID-19 vaccine. Among them, 8930 children had completed the primary vaccination series, while 50 children had obtained a booster dose [2].

The existing data indicate that a minimum of one-third of SARS-CoV-2 infections manifest as asymptomatic cases [4]. Longitudinal investigations further suggest that approximately 75% of individuals who test positive for SARS-CoV-2 via PCR testing, yet exhibit no symptoms at the time of testing, will persist in an asymptomatic state [4].

The prevalence of antibodies against SARS-CoV-2 is subject to notable fluctuations, showing regional and temporal variations [5]. The variability of serum markers is contingent upon several determinants in COVID-19, including the emergence of novel viral strains, the degree of viral transmission within communities, the implementation of public health interventions, modifications in vaccination strategies, and the level of prior natural infection within the population [6]. In the context of children, the magnitude of seroprevalence is influenced by factors such as their age, gender [7], localized rates of viral transmission, and the extent of diagnostic testing and surveillance efforts [8].

Empirical seroprevalence surveys have demonstrated the susceptibility of children to SARS-CoV-2 infection. COVID-19 in children usually manifests with milder symptoms compared to adults, predominantly featuring asymptomatic presentations among pediatric cases [9]. The increased prevalence of asymptomatic infections in children has sometimes led to undetected infections.

Considerable uncertainties persist regarding the inherent characteristics and enduring nature of immunity subsequent to SARS-CoV-2 infection in pediatric populations. The available body of evidence, albeit restricted, elucidates the humoral and cellular reactions to the virus, revealing a heightened innate immune response in the initial stages of infection. This augmented innate immune response is posited to constrain the extent of the infection, consequently contributing to the favorable clinical outcomes observed in children [10].

An additional noteworthy consideration pertains to the prospect of the transmission of SARS-CoV-2 antibodies via breast milk, stemming from mothers who have undergone infection [11], received COVID-19 vaccination [12], or by transplacental transmission during pregnancy [13].

Children under the age of five are more likely to contract or transmit COVID-19 because they frequent community settings such as nurseries and kindergartens [14,15]. Additionally, many parents hesitate to vaccinate their children [16,17,18], and there is also a potential passive immunity against SARS-CoV-2 transmitted transplacentally or through breast milk.

For these reasons, we evaluated the extent of anti-SARS-CoV-2 IgG seroprevalence in children under 5 years old, for whom the vaccine received approval late in the course of the pandemic, and who frequently have mild symptoms or asymptomatic forms of the disease. We aimed to determine the SARS-CoV-2 seroprevalence of the aggregate of past infection, passive immunity and anti-COVID-19 vaccination. Hence, we assessed the presence of anti-SARS-CoV-2 protective antibodies in children below the age of five through the quantification of anti-S1 receptor-binding domain (RBD) immunoglobulin G (IgG) titer. 

To the best of our knowledge, a majority of SARS-CoV-2 seroprevalence investigations conducted in pediatric populations have predominantly focused on the mere presence of antibodies, omitting an in-depth analysis of their quantitative levels. In our study, we meticulously examined not only the prevalence of IgG antibodies against SARS-CoV-2, but also assessed the specific titers associated with these antibodies, thereby contributing a comprehensive perspective to the existing body of research in this domain.

## 2. Materials and Methods

### 2.1. Study Design and Participants

We conducted a descriptive cross-sectional study which aimed to measure the anti-SARS-CoV-2 IgG titer in serum samples of hospitalized children.

The inclusion criteria were as follows: admission to hospital, age between 0 and 5 years, consent of the patient’s legal guardian to participation in the study, and blood collection for usual analyses during hospitalization.

The exclusion criteria were as follows: SARS-CoV-2 positive patients, the presence of signs and symptoms that correlate with an acute respiratory infection, immunosuppressed or immunodepressed patients, and oncological patients. 

We included 743 consecutive patients admitted to the “Sfânta Maria” Children’s Emergency Hospital in Iași, Romania, between the 1 August 2022 and the 15 September 2023. Upon admission, all hospitalized individuals underwent testing using a lateral flow immunochromatographic assay designed for the identification of SARS-CoV-2 antigens. The patients were stratified into distinct subgroups according to their ages, delineated as follows: 0–12 months, 13–24 months, 25–36 months, 37–48 months, and 49–59 months. Demographic data (age, gender) and the reason for admission to hospital were collected for all patients. These data are available in the table from the Supplementary Materials.

### 2.2. Sample Collection and Analysis

Serum samples were obtained from each patient upon the receipt of informed consent from their legal guardian. These samples were extracted from blood collected for routine analyses, with approximately 200 μL of serum being transferred to Eppendorf tubes, subsequently frozen at −20 °C and stored for further analyses.

Reactive anti-S1 RBD IgG antibodies were quantified in all serum samples using a sandwich enzyme-linked immunosorbent assay (ELISA). Sample processing followed the manufacturer’s instructions (TestLine Clinical Diagnostics, Brno, Czech Republic, Catalog number: CoRG96-EIA COVID-19 RBD IgG, with reported test specificity of 99.15% and test sensitivity of 99.9%). The manufacturer-specified reactivity threshold for the anti-S1 RBD IgG titer was established at 18 U/mL according to the kit specifications. Optical densities were measured at a wavelength of 450 nm using a TECAN Infinite 200 photometer (Tecan, U.S., Inc., Morrisville, NC, USA), and the results were analyzed using the Magellan software (5th version).

Quantification of IgG levels was carried out on serum samples collected as part of routine diagnostic and monitoring procedures, essential for assessing the patient’s clinical evolution. Importantly, because the determination of anti-SARS-CoV-2 immune status was not dependent on the timing of serum sampling during the actual hospitalization, the moment of collection was not specifically predetermined for the study, but rather occurred opportunistically during routine healthcare practices. Consequently, the timing of serum collection was random, regardless of the date of hospitalization or onset of symptoms. Also, for the study purpose, the time of sampling during the child’s hospitalization was not important. This decision was motivated by the ethical imperative to minimize any additional distress or discomfort experienced by pediatric patients, who are already in a challenging emotional state due to the underlying illness that required their hospitalization.

### 2.3. Ethical Principles

This investigation adhered to the ethical tenets articulated in the World Medical Association’s Declaration of Helsinki concerning medical research involving human subjects. Ethical approval for the study was granted by the Ethics Commission of the “Sfânta Maria” Children’s Emergency Hospital in Iași, Romania (IRB number 13314/10.05.2022), and the Commission of Ethics of Research from “Grigore T. Popa” University of Medicine and Pharmacy in Iași, Romania (IRB number: 211/14.07.2022).

### 2.4. Statistical Analysis

The statistical analyses were conducted utilizing the 26th version of the IBM SPSS (IBM Corp. Released 2019. IBM SPSS Statistics for Windows, Version 26.0. Armonk, NY, USA: IBM Corp) statistical software. The distribution of variables was assessed by employing the Kolmogorov–Smirnov test. Correlations between variables were examined using Pearson or Spearman correlation tests. The *p*-value served as a metric for evaluating the α-significance level, where a level below 0.05 indicates a probability of less than 5% that the observed event occurred by chance. The α-significance level denotes the probability of encountering false positives. Correlation strength was delineated based on the r results: 0–0.29 indicated poor correlation, 0.3–0.49 signified a medium correlation, and 0.5–1 denoted a strong correlation. Group comparisons were executed using the one-sample test, independent samples test, and ANOVA test. Analyses were performed for both the entire study group and five distinct age subgroups. The discussion and conclusions drawn in the study were substantiated by the outcomes of the statistical tests. Descriptive statistics, including the mean, median, standard deviation, range, and percentiles of age and anti-SARS-CoV-2 antibody titers, were computed using the same statistical software.

## 3. Results

**Figure 1:**
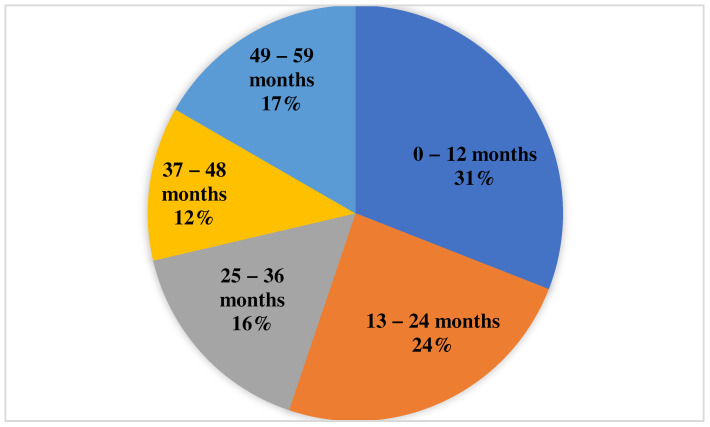
Distribution of participants by age group.

The study group comprised 743 participants aged 0–59 months, assigned into five subgroups based on their respective age categories, as illustrated in Figure 1. Specifically, there were 230 individuals in the 0–12 months’ subgroup, 180 in the 13–24 months’ subgroup, 120 in the 25–36 months’ subgroup, 89 in the 37–48 months’ subgroup, and 124 in the 49–59 months’ subgroup (Figure 1). The mean age was 22.64 months and the median was 21 months for the entire group (Table 1). Other important descriptive statistical parameters related to age can be seen in Table 1.

**Figure 2:**
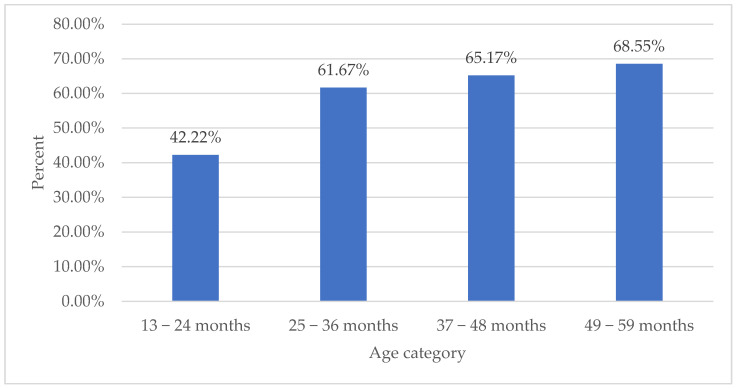
IgG prevalence by age category.

Of the study participants, the anti-SARS-CoV-2 IgG titer exceeded the kit reactivity threshold value in 392 (52.76%) of the patients. The mean anti-SARS-CoV-2 IgG titer within the entire research group was 1558.01 U/mL. Additional relevant descriptive statistical parameters associated with the anti-SARS-CoV-2 IgG titer are detailed in Table 1 and Figure 2, offering an overview of the different quantitative aspects and distributions of the measured IgG levels.

In the 0–12 months’ age group, 43.04% of children showed anti-SARS-CoV-2 antibodies, while in the age range of 13–24 months, the corresponding percentage was 42.22% (Figure 2, Table 2). Moving to the 25–36 months’ category, a notable 61.67% of sera were reactive in the anti-SARS-CoV-2 antibody detection, followed by the 37–48 months’ group with a prevalence of 65.17%. Finally, in the age range of 49–59 months, a majority of 68.55% demonstrated anti-SARS-CoV-2 seropositivity (Figure 2, Table 2).

**Figure 3:**
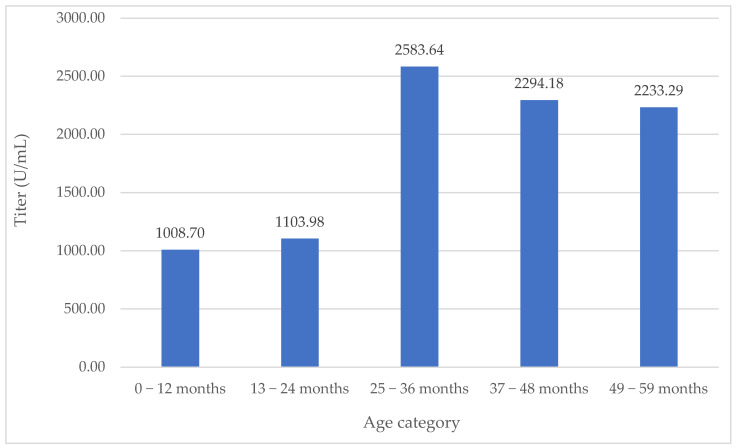
Mean anti-SARS-CoV-2 IgG titer (U/mL) depending on age category.

Mean anti-SARS-CoV-2 IgG titers by age group ranged from 1008.70 U/mL, documented in the 0–12 months’ age category, to 2583.64 U/mL, observed in the 25–36 months’ age category, as shown in Table 2 and Figure 3.

**Figure 4:**
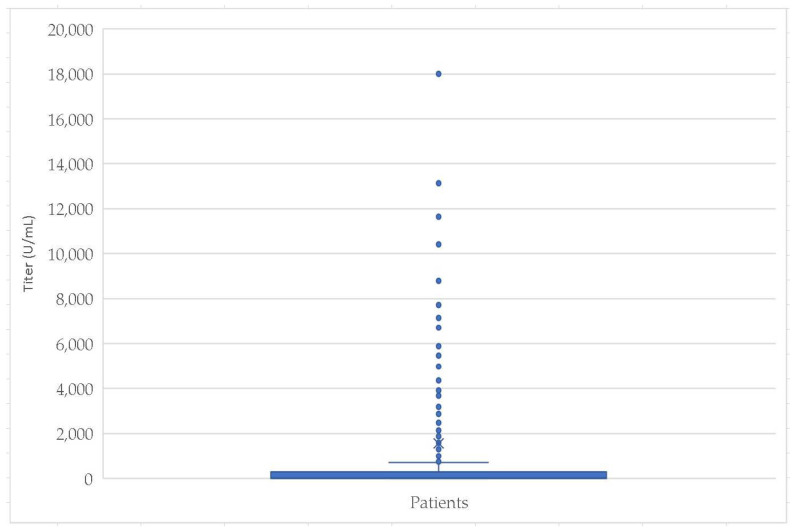
Anti-SARS-CoV-2 IgG titer (U/mL) in the entire sample.

**Figure 5:**
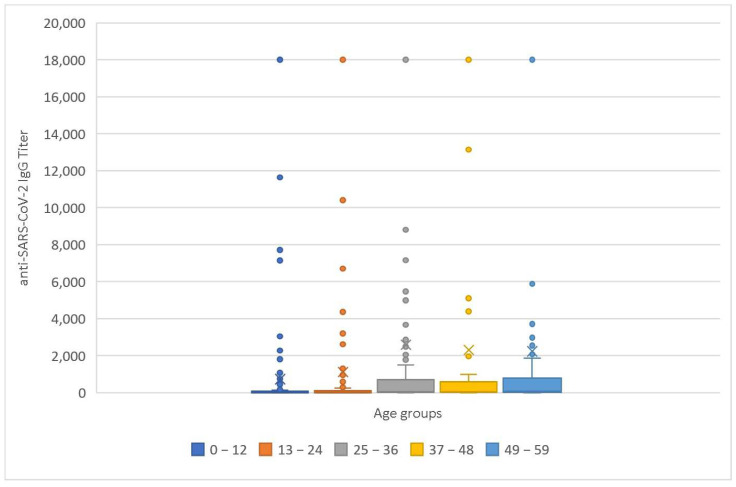
Anti-SARS-CoV-2 IgG titer (U/mL) depending on age category.

Figure 4 and Figure 5 depict the distributions of anti-SARS-CoV-2 antibody titers for the entire study group and the age categories, respectively.

Within the study cohort, a total of 411 individuals, representing 55.32% of the participants, were males, among whom the seroprevalence rate was established at 52.07%. Of the female subgroup, a noteworthy 54% exhibited anti-SARS-CoV-2 IgG titers surpassing the predefined threshold value (Table 2).

Approximately 40% of the patient population, 297 individuals, were subjected to emergency hospitalization. Thirty-two cases (4.3%) needed admission to the intensive care unit during their hospitalization (Table 2). Of the patient population, 449 individuals (60.4%) exhibited gastrointestinal disturbances, 34 (4.6%) manifested renal symptoms, 28 (3.77%) had dermatological disorders, and 39 (5.25%) were diagnosed with cardiac problems. The remaining cases included individuals with diverse other conditions such as food intolerances, infections, and surgical issues.

The relevant findings were elucidated using SPSS analyses, including either Pearson or Spearman correlation examinations. These analyses revealed direct correlations between the antibody titer levels and the age of the children (r = 0.195, *p* < 0.001). Moreover, direct correlations were observed within specific subgroups corresponding to distinct age categories (r = 0.228, *p* < 0.001) (Table 3).

The One-Sample Test analysis revealed a statistically significant increase in anti-SARS-CoV-2 IgG titer relative to the threshold value in the entire batch (*p* < 0.001). 

The Independent Samples Test revealed significant differences between the ages of the patients in terms of their seroprevalence status (Table 4). However, the same test did not identify any statistically significant difference in antibody titers, their prevalence, emergency room or intensive care admissions by gender (Table 5).

The One-Way ANOVA analysis demonstrated notable variations in anti-SARS-CoV-2 IgG titers among patients by age or age category (*p* < 0.001). In addition, significant differences were observed in terms of emergency hospitalization or transfer to intensive care depending on the patient’s age or age subgroup (*p* < 0.001).

## 4. Discussion

The strength of this study was that, to date, this is the first antibody-based epidemiological investigation of the seroprevalence of SARS-CoV-2 within the pediatric population of Romania, after the COVID-19 vaccine was approved for children. Moreover, it is one of the few studies that have thoroughly analyzed anti-SARS-CoV-2 IgG titers among children and their associations with various demographic factors worldwide.

As a comprehensive assessment, the results of the present study are of considerable epidemiological and public health significance, emphasizing the imperative of formulating evidence-based guidelines for early childhood establishments such as nurseries, kindergartens, or preschools, as well as other community activities relevant to early childhood. The increased seropositivity observed among children in this study provided valuable information in that it may provide a more accurate estimate of asymptomatic infections. This, in turn, was crucial to thoroughly assessing the extent of SARS-CoV-2 transmission, to gaining an insight into local outbreaks of the infection, and to developing effective strategies for future outbreak containment efforts.

WHO reports for the period between 30 December 2019 and 25 October 2021 indicated that 2% (1,890,756) of globally reported COVID-19 cases pertained to children under the age of five. Older children and younger adolescents (5 to 14 years) constituted 7% (7,058,748) of the global caseload, while older adolescents and young adults (15 to 24 years) accounted for 15% (14,819,320) of the reported cases globally [19].

In the United States, as of July 2022, 14,003,497 cases were documented in children, representing 18.6% (14,003,497/75,463,921) of all reported cases. Globally, by 24 July 2022, children under the age of five and those aged 5–14 constituted 2.47% and 10.44%, respectively [20]. 

In our country, according to the National Institute of Public Health’s report, as of 30 January 2024, there were 3,521,328 reported cases of SARS-CoV-2 infections. Approximately 5% of cases were recorded in children aged 0–9 years, around 7% in the group aged 10–19 years, roughly 11% in the group aged 20–29 years, about 16% in the group aged 30–39 years, approximately 18% in the group aged 40–49 years, and 15% in the group aged 50–59 years [2]. 

Reduced susceptibility to SARS-CoV-2 infection and attenuated symptomatology present substantial challenges in delineating the prevalence of pediatric infections. This highlighted the critical role of serological assessments in understanding the impact of the pandemic. 

Pre-existing cross-immunity stemming from prior exposure to endemic coronaviruses could confer a degree of protective immunity against SARS-CoV-2 [14]. Nonetheless, older adults showed high levels of cross-reactive IgA and IgG antibodies against SARS-CoV-2 in comparison to children, whose predominant antibody response is characterized by IgM [15]. This observation implied a less experienced yet more reactive immune response in children, which may be attributable to their limited exposure to endemic coronaviruses [14]. The extent to which cross-immunity contributed to the attenuation of disease severity in children remains uncertain. Given the discernible divergence in infection rates among school-aged children, the acquisition of age-stratified data was imperative to understand variations in the immunological response to SARS-CoV-2. Such insights were essential in elucidating strategies aimed at safeguarding the pediatric population [9].

Cases of COVID-19 infection in the pediatric population have attracted considerable attention, given the inherent vulnerability often observed in this age group. Despite this, the clinical presentation of the disease in children is typically less severe in comparison to adults, with a notable prevalence of asymptomatic cases among pediatric cases of COVID-19. The propensity for asymptomatic infections has sometimes led to cases where children have unknowingly endured infection [8,9].

It is known that anti-SARS-CoV-2 IgG can be transmitted through breast milk or transplacentally; therefore, another relevant aspect worth considering concerns the possible transmission of passive immunity to infants through the mechanism of breastfeeding, particularly from mothers who had either contracted SARS-CoV-2 [11] or received vaccination against COVID-19 [12].

Research conducted subsequent to October 2021 on COVID-19 vaccines for children aged 5–11 revealed a notable prevalence of parental vaccine hesitancy and limited uptake. This highlighted the imperative need to understand the attitudes and beliefs that drive parental rejection or acceptance of COVID-19 vaccines for younger children. Parental intentions to vaccinate children aged 1–4 years against COVID-19 were influenced by various factors, including education, financial stability, maternal vaccination history, COVID-19 health beliefs, safety and efficacy concerns, and community and family support. Notably, Fisher et al. (2022) found a positive correlation between higher levels of education, income, and financial security with increased vaccine acceptance, underscoring the impact of unclear messages regarding safety and efficacy [16]. 

Considering the data provided by the National Center for Supervision and Control of Communicable Diseases in Romania, indicating that only 0.22% of children are fully vaccinated, it can be concluded that in our region, parents are opting against vaccinating their children for COVID-19 [2].

A cross-sectional investigation conducted by Pistol et al. in 2021, with the objective of evaluating the prevalence of SARS-CoV-2 antibody seropositivity in Romania, revealed a seroprevalence rate of 8.02% in the north-eastern region of the country at the time of the study. Furthermore, within the study group of children aged 0 to 9 years, a seropositivity rate of 6.1% was documented in the same region [21]. 

The current research encompassed a cohort of 743 pediatric patients under 5 years of age who were admitted to “Sfânta Maria” Children’s Emergency Hospital in Iași, Romania. In particular, individuals who had been hospitalized for acute respiratory conditions, oncological diseases, or patients with comorbidities associated with immunosuppression were excluded from the study.

We made a deliberate decision to exclude individuals with cancer, immunodepressed individuals, or those undergoing immunosuppressive treatment from the study. This choice was motivated by the intention to mitigate any potential impact on their immune response, thereby avoiding the risk of obtaining a false-negative result in terms of antibody prevalence. The exclusion of this patient subgroup was necessary to maintain the accuracy and reliability of the study results, ensuring that the observed antibody prevalence remains representative of the broader population under investigation. It has been established in the medical literature that individuals undergoing complete immunosuppression face an elevated risk of delayed antibody response and unfavorable clinical outcomes [22]. This assertion was also supported by the findings of Minotti et al. (2020) [23], who concluded that immunosuppressed patients, predominantly including those with cancer, as well as transplant recipients and those with immunodeficiency, exhibit a diminished immune response characterized by weakened antibody production.

Furthermore, patients with acute respiratory infections were excluded from the study protocol. This precautionary measure aimed to mitigate the potential confounding factor of newly acquired cases of COVID-19, wherein the manifestation of an immune response, as objectified by IgG, may not yet have occurred. This exclusion criterion was implemented to enhance the precision and validity of the study outcomes by focusing on individuals with pre-existing immunity or those who had recovered from prior infections, but not acute ongoing infections.

While the study was constrained in terms of the patient batch, focusing on “Sfânta Maria” Children’s Emergency Hospital, it is imperative to underscore that this hospital is a regional university facility catering to pediatric cases originating from the entirety of the north-eastern region of Romania.

The increased representation of the under 2 years’ age group could be attributed to more frequent hospitalizations of younger children, making them likely to participate in the study. Additionally, they have increased susceptibility to infections resulting from failure to comply with sanitary measures (e.g., improper wearing of masks). Furthermore, their attendance at operational nurseries and kindergartens during the peak of the epidemic, coupled with the absence of vaccination against SARS-CoV-2, contributed to their increased vulnerability. Additionally, the high risk of infectious diseases in young children is associated with the immaturity of their immune systems.

The demographic characteristics observed in this study underscored the prevalence of younger age groups and male representation within the studied pediatric population.

Within our study cohort, seroprevalence was 52.76%. The mean anti-SARS-CoV-2 IgG titer for the entire study group was 1558.01 U/mL (vs. 18 U/mL, the reactivity threshold), serving as a collective metric for the humoral immune response (Table 3). The different mean values across age groups in the study contributed to a nuanced understanding of the quantitative variations in humoral immune responses between different age groups within the study population. These averages marked the trend of anti-SARS-CoV-2 titers measured within the respective age groups, offering a quantitative representation of the humoral immune response to SARS-CoV-2 in different age strata.

Upon juxtaposing the results of our investigation with comprehensive international studies assessing the seroprevalence of SARS-CoV-2 in pediatric populations, it became evident that the seropositivity rate identified in our study surpasses most of the previously documented global figures, particularly in the first year of the pandemic, ranging between 1% and 15% [24,25,26,27,28,29,30,31,32,33] (Table 6). The discernible disparity observed can be elucidated by the considerable impact of several factors on seropositivity, encompassing divergences in the temporal and environmental contexts of the studies, sociodemographic attributes characterizing the study participants, varying levels of exposure risk, and the implementation of measures such as school closures.

In 2021, a research endeavor conducted in western Romania underscored a notable seroprevalence of SARS-CoV-2 among children, reaching 46.70%. Specifically, within the subset of preschool-aged children (1–5 years), a prevalence rate of 39.81% was documented [41].

The high prevalence observed in the present study can be explained by the timing of the study, as the investigation was conducted within a timeframe between 30 and 42 months after the first documented case of COVID-19.

The seropositivity rate objectified by us closely paralleled the rates documented in other Central European nations, as exemplified by Franczak et al. (2022), particularly in the case of Poland [36].

Our findings revealed a notable prevalence of humoral immune response within the specific demographic subset of girls. The observed variations in seroprevalence between male and female participants contributed valuable insights into the differential patterns of humoral immune response within the studied population according to gender. 

Despite the absence of statistical significance in the observed disparity between the genders, the elevated seropositivity rate identified among female participants may be attributed to the gender-based differences in immune responses. Moreover, the enhanced antibody response post-vaccination [42] or post-infection [43] in females compared to males aligns with established patterns in gender-based differences in immune responses. Moreover, females exhibit a comparatively lower susceptibility to viral infections than males, owing to distinctions in innate immunity and factors associated with sex chromosomes. The presence of two X chromosomes in females accentuates the immune system’s robustness, even when one X chromosome is transcriptionally inactive. The immune regulatory genes encoded by the X chromosome in the female gender contribute to diminished viral load levels and reduced inflammation compared to males. Concurrently, females manifest elevated levels of CD4+ T cells, indicative of a more robust immune response. Moreover, women typically generate higher antibody levels that persist in circulation for extended durations [44]. 

The findings derived from our study were backed up by the results documented by Mustafa et al. (2020), as elaborated in their systematic review [45]. The results of the latter indicated no statistically significant differences in the incidence of COVID-19 according to the gender of pediatric patients [46]. Our findings were congruent with those reported by other researchers in the field [7,35,47,48].

The clinical presentation of the patients revealed a diverse spectrum of manifestations, encompassing various organ systems. Moreover, it illustrated the multifaceted nature of the observed clinical conditions and contributed to the complexity and heterogeneity of the clinical profile within the study population. Each patient admitted for gastrointestinal pathology received confirmation of a diagnosis other than COVID-19 during their hospitalization.

The use of Pearson and Spearman correlation tests provided valuable insights into the complex relationship between age, seroprevalence, and antibody titer in the examined pediatric cohort (Table 3). These correlations confirmed the observation that antibody titers increase with increasing age in children. In addition, the dependence of seroprevalence on age groups has become evident, indicating an increased seroprevalence in older age groups. This comprehensive analysis has improved our understanding of the interaction between age, seroprevalence, and antibody titers in the context of the pediatric population under investigation.

The one-sample test indicated a statistically significant increase in antibody titers compared to the kit threshold value. This observed increase was found to be statistically significant for the entire study group, as indicated by a *p*-value less than 0.001. The significance of this finding underscored the substantive deviation from the baseline threshold value within the studied population.

Comparison tests elucidated that as the age of patients increased, there was a corresponding increase in their seropositivity rate (Table 4). This discernible association suggested a trend where older patients demonstrated a higher proportion of SARS-CoV-2 antibody detection, elucidating a potential age-dependent pattern in the acquisition of immune protection. Asseri et al. (2022) also observed that the SARS-CoV-2 seroprevalence was conditional on the age of the child, a correlation that is reinforced by the results of our study [7]. Franczak et al. (2022) observed, as we did, that antibody titers were higher in older children than in younger children [36]. Similar findings have been highlighted by other researchers as well [35,47,48,49]. 

Our study was restricted by its hospital-based design. Limitations arose from the unavailability of additional patient characteristics that could have extended correlation analyses with other variables. The data collection process might also have been subject to bias, as it relied on information obtained from medical records. Given these constraints, we recommend the implementation of community-based studies to more accurately estimate the seroprevalence rate among the pediatric population in the country. In addition, it is essential to acknowledge that the presence of antibodies may decrease over time; thus, some children who contracted SARS-CoV-2 early in the pandemic may not have tested positive due to antibody levels that became undetectable.

## 5. Conclusions

More than half of the tested children had SARS-CoV-2 anti-S-RBD IgG. The anti-SARS-CoV-2 IgG titer increased with the child’s age. Gender, admission diagnosis, and emergency admission were determined not to be significant factors influencing variations in the IgG titer.

## Figures and Tables

**Table 1:** Descriptive statistics calculated for the age of the participants and anti-SARS-CoV-2 IgG titer.

Statistic Parameters		Age (Months)	Titer (U/mL)
Mean		22.64	1558.01
Std. Error of Mean		0.60	167.77
Median		21.00	22.90
Std. Deviation		16.32	4573.00
Range		57.80	18,000.00
Minimum		0.20	0.00
Maximum		58.00	18,000.00
Percentiles	25	9.00	6.91
	50	21.00	22.90
	75	36.00	297.10

**Table 2:** Characteristics of each age group.

Age Group (Months)	Sample Size (Patients)	Mean IgG Titer (U/mL)	Median IgG Titer (U/mL)	Mean Age (Months)	Median Age (Months)	Males	Emergency	Admission to ICU
0–12	230	1008.70	12.18	5.76	6	51.74%	38.7%	7.4%
13–24	180	1103.98	11.47	16.91	16	56.11%	42.35%	4.4%
25–36	120	2583.64	62.21	30	30	55.83%	37.5%	5%
37–48	89	2294.18	54.8	42	42	57.3%	43.8%	0%
49–59	124	2233.29	63.5	54	54	58.87	41.94%	0.8%

Abbreviation: IgG = immunoglobulin G; ICU = intensive care unit.

**Table 3:** Correlation parameters revealed by Pearson and Spearman tests.

Parameters	Seroprevalence	Ab Titer
Age	***p*** ** < 0.001**	***p*** ** = 0.001**
Age Group	***p*** ** < 0.001**	***p*** ** < 0.001**
Gender	*p* = 0.675	*p* = 0.337
Diagnosis	*p* = 0.692	*p* = 0.743

Values in bold are statistically significant. Abbreviations: *p* = statistical coefficient; Ab = antibody.

**Table 4:** Significant differences between variables depending on the seroprevalence status.

Parameters	Age	Age Group	Diagnosis	Gender
Anti-SARS-CoV-2 IgG Seroprevalence	***p*** ** < 0.001**	***p*** ** < 0.001**	*p* = 0.692	*p* = 0.675

Values in bold are statistically significant. Abbreviations: IgG = immunoglobulin G; *p* = statistical coefficient.

**Table 5:** Significant differences between variables depending on gender.

Parameters	Seroprevalence	Ab Titer	Emergency Room Admission	ICU Admission
Gender	*p* = 0.337	*p* = 0.330	*p* = 0.729	*p* = 0.729

Abbreviations: Ab = antibody; *p* = statistical coefficient.

**Table 6:** Comparisons between our results and those of other authors.

Results	Seroprevalence	Study Period	Sample Size	Geographical Area	Age (Years)	Mean Titer	Anti-SARS-CoV-2 Ab Type
Our study	52.76%	1 August 2022–15 September 2023	743	Romania	0–5	1559.01 U/mL	anti-S
Sapronova et al. (2023) [34]	77.80%	March–July 2022	81	Latvia	<5		anti-N and anti-S
Naeimi et al. (2022) [35]	11.01%	December 2021–July 2022	42,889	Review	<5		NA
Franczak et al. (2022) [36]	57% (69% being < 5 y)	1 June 2021–30 April 2022	272	Poland	<18	539.85 (BAU/mL)	anti-S
Clarke et al. (2023) [37]	68%	February 2022	6096	USA	1–4		anti-N
Asseri et al. (2022) [7]	36%	1 October 2021–30 November 2021	246	Saudi Arabia	1–5		NA
O’Brien et al. (2022) [38]	39%	12 May 2021–13 July 2021	254	USA	1–4		anti-N and anti-S
Zinszer et al. (2021) [26]	4.82%	22 October 2020–22 March 2021	1632	Canada	2–4		anti-S
Elton et al. (2023) [39]	21%	11 January 2021–14 March 2021	871	Switzerland	0–5		anti-S
Comar et al. (2021) [27]	9.50%	5 January 2021–31 January 2021	169	Italy	<18	482.3 ± 387.1 BAU/mL	anti-S
Cavalcante Pinto Junior et al. (2021) [29]	22.70%	9 November 2020–9 December 2020	423	Brazil	<9		anti-N
Torres et al. (2020) [40]	12.30%	4 May 2020–19 May 2020	1009	Chile	<5		NA

Abbreviations: y = years; NA = not available.

## Data Availability

The data presented in this study are available on request from the corresponding author.

## Supplementary Materials

The following are available online at https://www.mdpi.com/article/10.3390/medicina60030384/s1, Table S1: Patients’ data.
