# Supplementary material for: SARS-CoV-2 Seroprevalence in Children under 5 Years Old—A Regional Seroepidemiological Study
Source: Medicina (Kaunas). 2024 Feb 24;60(3):384. doi: 10.3390/medicina60030384 (PMC10972281; doi:10.3390/medicina60030384)
Supplement: Supplementary file 1 [file medicina-60-00384-s001.zip › medicina-2825022-supplementary.pdf]

No	Age (months)	Age group	Gender	Disease category	Seroprevalence	Titer
1	37	4	1	1	1	137.44
2	3	1	1	2	2	8.40
3	37	4	1	2	2	13.52
4	10	1	2	3	2	0.00
5	0.3	1	1	1	1	578.82
6	21	2	1	4	1	497.88
7	37	4	2	4	2	10.01
8	14	2	2	2	1	45.61
9	8	1	1	2	1	32.88
10	37	4	2	2	2	0.00
11	58	5	2	5	1	517.65
12	25	3	1	3	2	0.00
13	49	5	2	4	2	7.45
14	13	2	2	2	2	5.31
15	25	3	2	5	1	468.71
16	25	3	2	2	2	16.16
17	25	3	1	1	1	49.19
18	6	1	1	1	1	118.97
19	58	5	1	1	2	7.94
20	20	2	1	3	2	8.13
21	25	3	2	2	1	18000.00
22	21	2	1	5	1	18000.00
23	22	2	1	6	1	68.85
24	6	1	2	3	2	17.34
25	20	2	1	2	1	252.31
26	3	1	1	7	2	7.64
27	25	3	1	3	1	54.34
28	25	3	1	3	2	7.48
29	4	1	2	2	1	286.15
30	1	1	2	7	1	867.71
31	37	4	1	2	1	506.35
32	0.2	1	2	5	1	420.71
33	12	2	1	2	2	5.11
34	10	1	2	2	2	5.51
35	11	1	1	2	2	5.38
36	22	2	2	2	1	43.93
37	11	1	1	2	2	8.52
38	25	3	1	4	1	18000.00
39	11	1	2	2	1	62.75
40	58	5	1	2	1	18000.00
41	37	4	2	2	1	21.95
42	58	5	2	3	1	63.59
43	6	1	1	3	2	12.19
44	6	1	2	5	1	39.93
45	37	4	1	7	1	18000.00
46	17	2	2	7	2	5.73

47	25	3	2	7	2	7.75
48	49	5	1	2	2	11.19
49	37	4	2	1	2	7.25
50	23	2	1	3	1	476.23
51	49	5	2	1	2	13.14
52	25	3	1	1	1	18000.00
53	25	3	2	2	1	607.06
54	37	4	2	1	1	18000.00
55	10	1	2	2	2	5.04
56	9	1	1	2	2	8.97
57	25	3	2	5	1	292.31
58	37	4	1	6	2	10.96
59	58	5	1	1	2	5.00
60	37	4	1	1	1	157.95
61	37	4	1	1	1	35.09
62	25	3	1	6	2	9.20
63	49	5	2	1	1	18000.00
64	49	5	2	1	1	29.20
65	20	2	1	1	1	28.89
66	25	3	2	1	1	28.26
67	16	2	2	1	2	5.38
68	49	5	2	3	2	17.19
69	17	2	2	1	2	5.99
70	15	2	1	1	2	6.68
71	12	2	1	1	2	8.63
72	14	2	2	1	2	11.84
73	49	5	1	1	1	47.71
74	11	1	1	5	1	27.63
75	25	3	1	1	2	14.17
76	6	1	1	1	2	6.91
77	25	3	1	1	1	18000.00
78	11	1	2	1	2	5.23
79	21	2	1	1	2	5.76
80	25	3	2	1	2	15.82
81	37	4	2	1	1	37.41
82	1	1	2	1	1	20.79
83	49	5	1	1	1	541.18
84	49	5	2	1	1	19.33
85	11	1	2	1	1	18.91
86	11	1	1	3	2	6.11
87	7	1	2	1	1	7140.00
88	14	2	2	1	2	5.31
89	25	3	1	5	2	7.40
90	6	1	2	5	1	35.84
91	13	2	1	1	2	7.56
92	14	2	1	1	2	0.00
93	37	4	1	1	1	505.00

94	0.7	1	2	1	2	7.07
95	37	4	1	1	2	14.54
96	3	1	1	1	1	58.96
97	9	1	2	1	2	5.49
98	17	2	2	1	2	5.41
99	1	1	2	1	1	533.00
100	18	2	2	1	2	0.00
101	6	1	2	1	2	6.79
102	8	1	1	1	2	5.85
103	4	1	2	2	2	10.60
104	16	2	2	1	1	41.86
105	3	1	1	1	2	9.26
106	1	1	1	1	1	80.84
107	25	3	1	1	1	23.45
108	13	2	2	1	2	0.00
109	49	5	1	1	1	18000.00
110	21	2	1	5	1	18000.00
111	9	1	2	1	1	45.92
112	7	1	2	1	2	10.40
113	4	1	2	1	1	54.69
114	3	1	2	1	2	10.07
115	49	5	1	1	1	19.45
116	4	1	2	1	1	29.81
117	25	3	1	1	2	6.99
118	2	1	2	1	1	69.70
119	37	4	1	1	1	71.12
120	21	2	2	7	2	7.44
121	5	1	2	1	2	6.30
122	25	3	1	1	2	10.16
123	14	2	2	1	2	0.00
124	16	2	1	1	2	12.84
125	5	1	1	1	2	5.20
126	49	5	2	1	1	24.55
127	37	4	2	1	2	6.10
128	19	2	1	1	1	25.10
129	1	1	2	5	1	18000.00
130	12	2	1	1	2	6.50
131	37	4	1	1	1	657.80
132	13	2	2	6	1	424.00
133	49	5	2	1	2	17.75
134	18	2	1	5	2	8.00
135	25	3	2	1	1	186.95
136	19	2	1	1	2	12.55
137	49	5	2	7	2	6.87
138	6	1	1	1	2	9.51
139	15	2	2	2	2	5.73
140	0.5	1	1	2	1	18000.00

141	3	1	2	2	2	7.11
142	3	1	1	2	1	18000.00
143	8	1	2	1	2	7.96
144	2	1	2	1	1	32.99
145	25	3	1	5	1	23.78
146	4	1	1	1	2	9.63
147	11	1	2	2	2	0.00
148	0.3	1	1	5	1	18000.00
149	6	1	2	2	2	5.57
150	10	1	2	2	2	7.72
151	49	5	1	2	2	14.42
152	37	4	1	6	1	39.34
153	49	5	2	6	1	551.00
154	10	1	1	1	2	5.20
155	10	1	1	1	2	0.00
156	13	2	2	1	1	18.60
157	25	3	1	2	1	71.23
158	3	1	1	1	2	13.40
159	10	1	1	2	1	146.53
160	49	5	1	2	1	18000.00
161	15	2	1	6	1	18000.00
162	25	3	2	6	1	18000.00
163	49	5	1	6	1	18000.00
164	8	1	2	1	2	7.80
165	25	3	1	1	2	8.21
166	49	5	1	1	1	389.00
167	8	1	2	1	2	0.00
168	14	2	2	7	2	5.89
169	25	3	2	6	2	8.21
170	19	2	1	6	1	629.00
171	37	4	1	1	2	7.35
172	25	3	1	7	1	67.84
173	16	2	2	1	2	14.70
174	2	1	1	1	1	629.00
175	25	3	1	2	2	5.93
176	49	5	1	2	2	6.34
177	5	1	2	6	2	9.20
178	0.25	1	2	5	2	12.84
179	7	1	1	1	2	6.42
180	6	1	1	1	1	29.06
181	25	3	1	2	2	17.99
182	37	4	1	2	1	106.57
183	10	1	1	1	2	6.37
184	7	1	1	1	2	5.47
185	15	2	1	1	2	6.84
186	25	3	2	1	1	42.43
187	23	2	2	2	2	8.02

188	7	1	1	1	2	5.71
189	20	2	2	1	1	601.35
190	25	3	2	7	1	604.94
191	13	2	1	1	2	8.50
192	25	3	1	6	2	12.04
193	7	1	2	1	2	0.00
194	37	4	2	1	1	18000.00
195	25	3	2	5	2	5.33
196	15	2	1	2	2	6.13
197	2	1	1	7	2	7.79
198	37	4	2	2	1	18000.00
199	25	3	1	1	1	18000.00
200	25	3	1	1	1	18000.00
201	4	1	1	2	2	5.57
202	14	2	2	2	2	7.74
203	9	1	1	2	1	297.10
204	15	2	1	6	2	7.13
205	49	5	1	1	1	18000.00
206	49	5	1	1	1	18000.00
207	4	1	2	5	2	12.28
208	11	1	2	2	2	12.09
209	14	2	1	2	1	20.43
210	37	4	1	2	1	18.84
211	1	1	1	6	2	15.82
212	22	2	2	2	1	18000.00
213	22	2	2	2	2	7.74
214	2	1	2	6	1	38.42
215	25	3	1	2	1	74.41
216	22	2	1	6	1	18000.00
217	15	2	1	6	2	0.00
218	49	5	2	6	2	0.00
219	6	1	1	2	2	0.00
220	49	5	1	1	2	0.00
221	22	2	1	5	2	0.00
222	25	3	1	2	2	0.00
223	25	3	1	2	2	0.00
224	7	1	1	2	2	0.00
225	19	2	1	2	2	13.79
226	37	4	1	2	1	380.23
227	14	2	1	2	2	11.09
228	4	1	1	2	2	9.20
229	25	3	1	2	2	8.92
230	12	2	2	2	2	5.80
231	25	3	2	2	1	284.27
232	12	2	2	7	2	5.47
233	49	5	1	2	1	105.65
234	1	1	1	5	1	795.39

235	49	5	1	2	2	17.24
236	37	4	1	7	1	22.01
237	49	5	1	1	1	18000.00
238	25	3	1	2	2	16.81
239	4	1	1	1	2	6.84
240	23	2	1	1	2	5.52
241	25	3	1	7	1	18000.00
242	9	1	1	7	2	5.28
243	37	4	1	7	1	18000.00
244	1	1	1	5	1	546.52
245	49	5	2	2	2	6.13
246	49	5	1	2	1	31.13
247	6	1	2	1	2	9.49
248	4	1	1	1	2	6.28
249	37	4	2	1	2	6.28
250	1	1	2	1	1	309.01
251	19	2	1	6	2	7.27
252	3	1	2	1	1	27.23
253	49	5	1	1	1	1000.00
254	49	5	2	1	1	1000.00
255	1	1	1	6	2	15.45
256	49	5	2	7	2	10.43
257	18	2	1	1	1	18.98
258	25	3	2	1	2	9.20
259	9	1	1	1	2	6.09
260	37	4	1	2	1	760.00
261	49	5	2	1	1	23.95
262	16	2	2	1	1	31.73
263	25	3	2	1	2	15.49
264	0.5	1	1	1	1	755.39
265	25	3	1	7	2	12.90
266	37	4	1	1	1	712.34
267	25	3	2	1	1	515.90
268	10	1	1	1	2	11.97
269	37	4	2	1	1	283.36
270	15	2	2	1	2	5.18
271	37	4	1	1	2	12.94
272	13	2	2	1	2	5.93
273	3	1	1	1	1	523.88
274	25	3	2	2	1	354.57
275	0.5	1	2	1	1	284.27
276	0.7	1	2	5	1	588.59
277	25	3	2	2	1	83.66
278	16	2	2	2	2	7.31
279	17	2	1	5	2	11.57
280	25	3	2	5	2	15.47
281	1	1	2	2	1	19.78

282	10	1	1	6	1	557.69
283	9	1	1	5	1	18000.00
284	4	1	1	5	2	9.79
285	25	3	2	6	2	12.50
286	25	3	1	2	1	18000.00
287	25	3	1	2	1	18000.00
288	25	3	2	1	1	165.19
289	25	3	2	2	1	75.84
290	2	1	1	2	1	781.31
291	12	2	2	2	1	58.20
292	37	4	1	2	2	5.40
293	25	3	2	2	1	46.69
294	8	1	1	2	2	5.71
295	21	2	1	2	1	25.51
296	9	1	1	2	2	12.19
297	10	1	2	5	2	5.22
298	4	1	2	1	1	19.87
299	2	1	2	1	2	13.65
300	49	5	2	1	1	49.14
301	11	1	1	1	2	0.00
302	15	2	1	5	2	5.53
303	19	2	2	1	2	6.29
304	37	4	1	1	2	6.82
305	37	4	2	7	1	18000.00
306	58	5	2	1	2	15.61
307	11	1	2	1	2	0.00
308	16	2	1	1	2	5.84
309	49	5	1	1	1	458.28
310	22	2	1	1	1	76.08
311	12	2	1	1	2	0.00
312	7	1	1	1	2	5.22
313	14	2	2	1	2	8.51
314	58	5	2	2	1	27.71
315	23	2	1	5	2	5.09
316	37	4	1	2	1	18000.00
317	8	1	1	1	2	9.84
318	20	2	1	5	2	6.95
319	58	5	2	2	1	18000.00
320	8	1	1	6	2	6.46
321	58	5	1	6	1	18000.00
322	22	2	2	7	2	0.00
323	17	2	1	2	2	15.25
324	14	2	2	1	2	9.08
325	20	2	2	1	2	8.15
326	49	5	2	2	2	5.98
327	7	1	2	2	2	5.53
328	37	4	1	2	2	6.82

329	49	5	1	2	1	64.57
330	13	2	2	2	1	47.06
331	3	1	1	7	2	7.84
332	14	2	2	2	2	6.07
333	58	5	2	1	1	29.92
334	17	2	1	2	2	5.98
335	9	1	1	7	2	8.86
336	6	1	2	2	1	45.35
337	25	3	1	2	2	6.78
338	49	5	2	7	1	848.60
339	11	1	1	1	1	777.94
340	9	1	2	6	2	8.02
341	25	3	1	7	1	37.02
342	49	5	1	6	2	17.16
343	58	5	1	3	2	0.00
344	49	5	1	2	1	55.27
345	9	1	2	2	2	10.28
346	23	2	2	1	2	17.52
347	7	1	2	3	2	0.00
348	49	5	2	3	1	556.68
349	37	4	2	3	2	17.47
350	25	3	2	2	1	359.00
351	49	5	2	2	1	19.20
352	10	1	1	2	2	8.11
353	8	1	1	2	2	5.87
354	37	4	1	6	2	13.78
355	37	4	1	3	2	9.58
356	37	4	2	3	1	810.12
357	17	2	1	4	2	8.55
358	58	5	1	1	2	7.67
359	14	2	2	2	2	8.27
370	3	1	1	1	1	36.74
371	2	1	1	1	2	7.24
372	49	5	1	1	1	789.27
373	21	2	2	1	1	217.55
374	37	4	2	1	2	17.33
375	37	4	2	1	1	315.26
376	1	1	2	1	1	865.00
377	25	3	1	1	1	678.41
378	37	4	1	1	1	637.48
379	21	2	1	1	2	6.42
370	20	2	2	1	2	13.07
371	9	1	1	1	2	0.00
372	1	1	1	2	1	755.24
373	25	3	1	1	1	78.07
374	18	2	1	1	2	6.53
375	10	1	2	1	2	6.53

376	8	1	2	1	2	5.65
377	17	2	2	1	2	7.89
378	11	1	2	1	2	7.29
379	37	4	2	1	1	143.56
380	25	3	1	3	1	207.12
381	12	2	1	1	1	39.26
382	49	5	1	3	1	18000.00
383	25	3	1	3	1	55.11
384	58	5	1	1	1	31.41
385	49	5	1	3	2	5.00
386	0.3	1	2	6	1	18000.00
387	0.4	1	2	2	2	6.20
388	6	1	1	1	2	8.38
389	25	3	2	1	1	78.37
390	14	2	1	1	2	9.85
391	3	1	2	6	1	48.00
392	14	2	1	6	1	960.49
393	15	2	2	7	2	9.09
394	12	2	2	4	2	7.07
395	15	2	2	1	1	18000.00
396	1	1	1	1	2	14.60
397	1	1	1	1	2	6.04
398	21	2	1	1	2	8.22
399	58	5	1	1	2	0.00
400	49	5	1	1	2	5.38
401	18	2	2	2	1	65.78
402	25	3	1	1	1	18000.00
403	25	3	2	1	1	59.26
404	37	4	2	1	1	42.22
405	16	2	2	1	2	0.00
406	23	2	1	3	2	0.00
407	58	5	1	1	1	28.44
408	8	1	2	1	2	7.36
409	37	4	1	1	2	17.00
410	58	5	1	7	2	5.55
411	21	2	1	1	1	588.35
412	25	3	1	2	2	6.91
413	13	2	1	1	2	12.80
414	2	1	2	1	1	419.53
415	49	5	2	2	1	71.41
416	58	5	2	1	1	31.85
417	6	1	1	1	2	0.00
418	49	5	1	1	1	330.08
419	22	2	2	1	2	14.87
420	58	5	2	1	2	6.31
421	37	4	1	1	2	6.85
422	3	1	1	2	2	17.65

423	10	1	2	2	2	0.00
424	11	1	2	4	2	5.60
425	3	1	1	1	1	25.19
426	8	1	2	4	2	5.05
427	21	2	2	7	2	0.00
428	58	5	1	4	1	203.32
429	25	3	2	1	2	0.00
430	9	1	2	1	2	0.00
431	58	5	2	1	1	18000.00
432	8	1	2	2	2	9.31
433	11	1	1	1	2	5.93
434	16	2	1	1	2	9.04
435	13	2	2	1	2	14.38
436	58	5	2	1	2	9.96
437	58	5	2	1	1	928.66
438	58	5	1	1	2	7.40
439	37	4	1	4	2	17.06
440	9	1	1	1	2	5.27
441	14	2	1	1	2	5.72
442	37	4	1	1	1	42.96
443	3	1	2	2	1	19.87
444	8	1	2	1	2	11.05
445	49	5	1	1	1	367.83
446	19	2	1	1	2	6.95
447	17	2	1	7	2	0.00
448	37	4	1	7	2	6.69
449	14	2	1	1	2	0.00
450	1	1	1	1	1	247.46
451	9	1	2	1	2	6.28
452	23	2	1	7	2	7.05
453	25	3	2	1	1	694.47
454	49	5	1	1	2	5.00
455	22	2	1	1	2	8.69
456	25	3	1	1	1	113.13
457	23	2	1	1	2	0.00
458	25	3	2	1	2	8.13
459	49	5	1	1	1	79.00
460	49	5	1	1	2	7.46
461	25	3	2	1	1	18000.00
462	1	1	2	4	2	0.00
463	25	3	1	1	2	6.54
464	25	3	1	1	2	0.00
465	19	2	2	7	2	0.00
466	23	2	2	7	2	0.00
467	23	2	1	7	2	0.00
468	25	3	2	1	2	0.00
469	7	1	2	7	2	0.00

470	10	1	1	7	2	0.00
471	18	2	1	2	2	0.00
472	49	5	1	3	2	0.00
473	10	1	1	2	2	0.00
474	58	5	2	4	2	0.00
475	25	3	2	4	2	0.00
476	49	5	1	3	2	0.00
477	37	4	1	2	2	0.00
478	1	1	2	7	2	0.00
479	9	1	2	2	2	5.72
490	13	2	1	2	1	79.57
491	16	2	2	2	2	0.00
492	25	3	2	2	2	0.00
493	11	1	2	2	2	5.87
494	8	1	1	1	2	0.00
495	49	5	2	2	2	0.00
496	37	4	2	2	2	0.00
497	37	4	1	2	2	0.00
498	8	1	2	2	2	0.00
499	14	2	2	1	2	0.00
490	13	2	2	4	1	18000.00
491	25	3	1	2	2	5.62
492	12	2	1	1	2	6.33
493	25	3	1	1	2	0.00
494	37	4	2	1	2	0.00
495	13	2	1	1	2	0.00
496	18	2	1	1	2	0.00
497	37	4	1	1	2	0.00
498	25	3	2	2	2	0.00
499	13	2	1	2	2	6.28
500	37	4	1	1	2	0.00
501	19	2	1	1	2	0.00
502	14	2	1	2	2	0.00
503	25	3	2	2	2	0.00
504	25	3	1	2	2	7.56
505	25	3	1	1	2	0.00
506	4	1	1	1	2	0.00
507	25	3	2	1	2	0.00
508	16	2	1	7	2	5.00
509	25	3	2	2	2	0.00
510	5	1	2	2	2	0.00
511	37	4	1	2	2	7.36
512	49	5	2	2	2	0.00
513	9	1	2	4	1	34.43
514	13	2	1	2	2	6.93
515	16	2	1	4	1	18.11
516	11	1	2	2	2	8.62

517	25	3	1	2	1	690.48
518	58	5	2	2	1	246.60
519	25	3	1	3	1	2124.00
520	58	5	2	3	1	2080.80
521	25	3	2	3	1	1773.36
522	0.7	1	1	5	1	453.78
523	37	4	2	7	1	180.72
524	20	2	1	2	2	5.90
525	16	2	1	4	2	8.48
526	58	5	1	2	1	875.34
527	14	2	1	2	1	4388.40
528	49	5	1	3	1	2966.40
529	13	2	2	3	1	2615.40
530	49	5	2	6	1	1866.60
531	2	1	1	6	2	3.01
532	10	1	2	7	1	740.70
533	37	4	1	7	1	830.16
534	4	1	1	4	1	90.85
535	15	2	1	7	1	59.94
536	37	4	2	7	1	331.92
537	1	1	2	4	2	5.20
538	7	1	1	7	1	66.37
539	17	2	1	2	1	3268.80
540	18	2	1	2	2	11.38
541	58	5	1	2	1	122.00
542	37	4	1	7	1	56.72
543	0.5	1	1	7	1	35.73
544	37	4	2	2	1	159.17
545	58	5	1	2	1	709.74
546	49	5	1	2	2	16.65
547	12	2	1	7	1	150.30
548	49	5	1	7	2	17.55
549	58	5	2	6	1	199.08
550	25	3	1	6	2	14.35
551	37	4	1	3	1	5160.60
552	37	4	1	2	2	3.83
553	49	5	1	2	2	13.70
554	25	3	1	2	1	751.50
555	58	5	1	3	1	105.89
556	49	5	2	3	1	242.28
557	13	2	2	7	1	19.04
558	7	1	2	7	1	23.90
559	58	5	1	2	2	8.96
560	17	2	1	2	1	106.16
561	58	5	1	4	1	319.86
562	7	1	2	6	2	12.17
563	5	1	1	6	2	9.50

564	37	4	1	2	1	272.16
565	49	5	2	3	1	915.66
566	37	4	1	3	1	22.90
567	37	4	1	2	1	811.08
568	3	1	1	2	1	353.52
569	9	1	2	4	1	24.12
570	25	3	2	2	1	7155.00
571	9	1	1	5	1	94.36
572	14	2	2	4	1	22.01
573	25	3	2	4	2	15.64
574	25	3	1	2	1	172.51
575	58	5	1	1	1	40.64
576	0.25	1	1	5	1	25.45
577	1	1	1	4	1	2273.40
578	25	3	1	2	1	5466.60
579	11	1	1	2	1	84.26
580	58	5	2	4	1	920.52
581	6	1	2	7	2	17.60
582	25	3	1	2	1	73.44
583	49	5	1	2	1	63.40
584	58	5	1	6	1	360.00
585	58	5	1	4	1	758.52
586	37	4	1	7	1	291.42
587	12	2	2	2	1	128.75
588	18	2	2	2	1	3191.40
589	11	1	2	2	2	11.03
590	16	2	1	2	1	1197.72
591	18	2	2	2	1	469.98
592	58	5	1	4	1	91.44
593	5	1	1	2	1	161.77
594	11	1	1	2	1	30.76
595	37	4	2	2	2	16.13
596	10	1	1	2	2	9.97
597	2	1	1	6	2	8.33
598	2	1	1	7	1	51.75
599	13	2	2	2	2	11.92
600	12	2	1	2	1	116.21
601	9	1	1	2	1	893.34
602	1	1	2	7	1	24.52
603	3	1	1	7	1	41.33
604	10	1	1	2	1	1067.94
605	1	1	1	2	1	31.45
606	22	2	2	2	1	84.98
607	7	1	2	2	1	41.65
608	25	3	1	2	1	530.64
609	14	2	2	2	1	151.87
610	2	1	2	2	1	1800.00

611	1	1	1	2	1	7704.00
612	19	2	2	2	1	78.95
613	25	3	2	2	1	4977.00
614	18	2	1	2	1	66.51
615	58	5	1	2	1	672.66
616	49	5	1	2	1	75.92
617	2	1	2	2	1	264.24
618	16	2	2	2	1	1297.98
619	4	1	1	2	1	24.12
620	6	1	1	1	2	16.07
621	58	5	2	1	1	5877.00
622	21	2	1	2	1	1077.84
623	6	1	1	2	1	189.72
624	21	2	1	2	1	1478.70
625	5	1	2	2	2	2.21
626	21	2	2	2	1	80.17
627	25	3	2	2	1	8794.80
628	1	1	2	7	1	279.36
629	37	4	1	1	1	27.41
630	25	3	2	1	1	32.72
631	25	3	1	4	1	2048.40
632	8	1	1	2	1	34.16
633	49	5	2	1	1	1334.34
634	5	1	1	1	1	40.66
635	25	3	1	1	1	2860.20
636	25	3	1	1	1	718.92
637	22	2	1	1	1	1082.88
638	15	2	1	1	1	3351.60
639	25	3	2	1	1	171.95
640	6	1	2	1	2	14.38
641	19	2	2	1	1	18000.00
642	49	5	2	1	1	1494.90
643	25	3	1	1	1	26.62
644	25	3	1	1	1	3670.20
645	7	1	1	1	2	9.74
646	49	5	2	2	1	1578.78
647	5	1	2	1	2	1.80
648	13	2	1	2	2	4.01
649	20	2	2	2	1	53.57
650	7	1	2	2	2	6.41
651	58	5	1	2	1	36.02
652	2	1	2	2	1	3038.40
653	25	3	2	1	1	347.94
654	37	4	2	7	1	4392.00
655	49	5	1	7	1	178.58
656	58	5	2	1	1	2545.20
657	49	5	1	1	1	19.62

658	37	4	2	1	1	51.86
659	11	1	1	1	1	1167.12
660	0.3	1	2	1	1	11633.40
661	2	1	1	2	1	1918.80
662	25	3	1	1	1	18000.00
663	49	5	2	1	1	335.52
664	49	5	1	1	1	19.39
665	6	1	2	1	2	14.62
666	37	4	1	7	1	18000.00
667	49	5	1	2	1	36.45
668	10	1	1	2	1	84.92
669	4	1	2	2	1	699.84
670	14	2	1	1	2	8.95
671	23	2	1	1	1	10405.80
672	13	2	1	2	2	12.51
673	17	2	2	2	1	286.92
674	25	3	1	2	2	9.85
675	14	2	2	2	1	63.32
676	17	2	2	2	1	6705.00
677	18	2	2	2	1	51.62
678	10	1	2	1	2	7.60
679	17	2	1	1	1	102.74
680	23	2	1	1	1	524.34
681	37	4	2	2	1	87.43
682	25	3	1	2	2	8.03
683	11	1	1	2	1	2512.80
684	11	1	1	2	2	3.35
685	37	4	2	1	1	54.79
686	12	2	2	1	1	4366.80
687	15	2	1	2	1	135.41
688	7	1	2	2	1	59.74
689	25	3	2	2	1	2480.40
690	37	4	2	2	1	386.64
691	7	1	1	2	1	123.80
692	37	4	2	2	1	306.36
693	19	2	2	1	2	3.17
694	37	4	2	2	1	18000.00
695	25	3	2	2	1	1487.34
696	37	4	2	2	1	13141.80
697	49	5	1	2	1	18.18
698	25	3	2	2	1	65.16
699	49	5	1	2	1	57.64
700	5	1	2	2	1	60.30
701	7	1	2	2	2	2.43
702	6	1	1	2	1	135.45
703	25	3	1	1	1	3920.40
704	37	4	1	1	1	5099.40

705	49	5	2	2	1	18000.00
706	6	1	2	7	1	57.49
707	25	3	1	2	1	18000.00
708	7	1	2	2	1	23.27
709	25	3	2	2	1	43.94
710	13	2	2	2	1	261.00
711	25	3	1	2	1	320.40
712	18	2	1	2	1	411.30
713	20	2	2	2	1	170.77
714	37	4	2	2	1	57.31
715	37	4	2	7	1	35.05
716	49	5	1	2	1	1768.86
717	4	1	2	2	2	1.80
718	4	1	2	2	2	9.22
719	2	1	1	2	2	1.80
720	25	3	1	1	1	160.58
721	25	3	2	1	1	35.01
722	49	5	1	7	2	3.31
723	25	3	2	2	1	130.97
724	6	1	1	2	1	37.78
725	21	2	1	7	1	108.90
726	3	1	1	2	2	12.60
727	37	4	2	1	2	1.80
728	37	4	1	1	1	1967.40
729	37	4	2	1	1	992.34
730	13	2	1	1	1	128.81
731	37	4	1	1	1	215.64
732	8	1	2	1	1	178.00
733	37	4	2	1	1	513.54
734	37	4	2	1	1	49.07
735	49	5	1	7	1	3704.40
736	13	2	1	2	1	108.94
737	25	3	1	2	1	191.70
738	49	5	1	1	1	465.66
739	49	5	2	1	1	101.32
740	49	5	2	1	1	88.70
741	19	2	2	7	1	227.34
742	16	2	1	2	1	71.08
743	9	1	1	1	2	3.80

Abbreviations:

Age group:	1=0-12 months
	2=13-25 months
	3=25-37 months
	4=37-49 months
	5=49-59 months
Gender:	1=male
	2=female
Disease category:	1=Emergency
	2=Gastroenterological
	3=Nephrological
	4=Dermatological
	5=ICU
	6=Cardiological
	7=Surgical
Seroprevalence:	1=positive
	2=negative
